# Eosinophilic cystitis in a patient with hypereosinophila syndrome: A case report

**DOI:** 10.3892/etm.2014.1706

**Published:** 2014-05-12

**Authors:** PENG JIANG, CHAOJUN WANG, BAIYE JIN, YIWEI LIN, SHANWEN CHEN

**Affiliations:** Department of Urology, The First Affiliated Hospital, School of Medicine, Zhejiang University, Hangzhou, Zhejiang 310003, P.R. China

**Keywords:** hypereosinophilic syndrome, eosinophilic cystitis

## Abstract

Hypereosinophilic syndrome (HES) is a rare disorder that is characterized by hypereosinophilia and organ damage, caused by the infiltration of eosinophils. In rare cases, the urinary bladder may also be involved. The current case report presented a 56-year-old male with gross hematuria and hypereosinophilia. The diagnosis of eosinophilic cystitis associated HES was established. Oral prednisone with a slow tapering regimen was administered as the primary treatment for the patient, which achieved partial hematological remission and complete relief of cystitis during a six-month follow-up period. Although eosinophilic cystitis is not commonly the primary manifestation of HES, eosinophilic cystitis should be taken into consideration following the onset of urinary symptoms in patients with HES.

## Introduction

Hypereosinophilic syndrome (HES) is a rare disorder that was first classified by Hardy and Anderson in 1968 (1). The syndrome refers to a group of leukoproliferative disorders that are characterized by the presence of marked peripheral blood eosinophilia and organ damage caused by the infiltration of eosinophils (2). HES used to be asympatic unless end organ injury due to massive eosinophilic invasivion happens. Virtually all organs can be involved, however, involvement of the urinary bladder is relatively rare. In the present study, a case of eosinophilic cystitis in a patient with HES was reported. To the best of our knowledge, there have only been a few definite cases previously reported in literature (3). Detailed clinicopathological data and follow-up information has been provided for the current case.

## Case report

**Figure 1:**
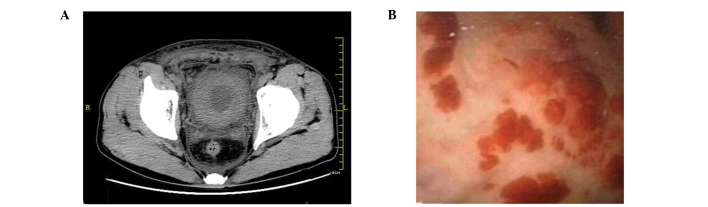
(A) Computed tomography image of the pelvis showing diffuse thickening of the bladder wall. (B) Cystoscopy observations revealed an erythematous velvety appearance of the bladder mucosa.

**Figure 2:**
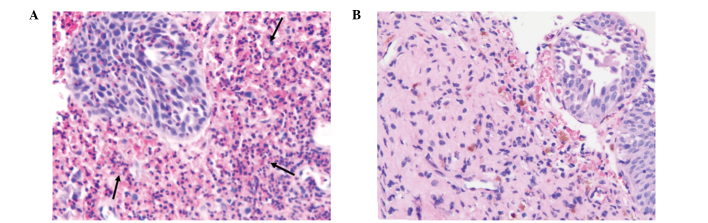
(A) Histological examination revealed dense interstitial eosinophilic infiltration in the bladder mucosa prior to prednisone therapy. (B) Eosinophilic infiltration was completely resolved following prednisone therapy (hematoxylin and eosin stain; magnification, ×200).

A 56-year-old male with one month history of gross hematuria and urinary urgency was admitted to our hospital on December 2012. The patient had no significant past medical history and the physical examination was unremarkable. Initial ultrasonography revealed marked thickening of the bladder wall, which was further confirmed by computed tomography scans (Fig. 1). Laboratory examinations revealed a white blood cell count of 16.8×10^3^ cells/mm^3^ (reference range, 4.0–10.0×10^3^ cells/mm^3^) and significant eosinophilia of 36% (reference range, 0.5–5.0%). Stool analysis for ova and parasites was negative. Cystoscopic examination was conducted and the observations revealed an erythematous velvety appearance of the bladder mucosa (Fig. 1). A biopsy was performed and the bladder mucosa showed diffuse infiltration of eosinophils, which indicated eosinophilic cystitis (Fig. 2). Bone marrow aspiration revealed marked eosinophilia, but no primitive cell predominance, which eliminated a diagnosis of leukemia. Therefore, hypereosinophilic syndrome (HES) complicated with eosinophilic cystitis was diagnosed and oral prednisone with a slow tapering regimen was administered to the patient for 6 weeks.

During the follow-up period of six months, the laboratory examinations revealed a fluctuant eosinophil count. At the most recent examination, the level of eosinophilia was shown to be 11%, which indicated a partial hematological remission. The subsequent cystoscopy and random bladder mucosa biopsies showed complete remission of cystitis histologically (Fig. 2).

## Discussion

HES is an uncommon condition characterized by eosinophilia and multiple organ damage. The disease has a significant male dominance and is usually diagnosed between the ages of 20 and 50 years (4). Currently, the pathophysiology of HES is not well described. The dysregulated overproduction of eosinophils may be due to a number of reasons, including the overproduction or dysfunction of eosinophilopoietic cytokines, such as interleukin-5, and clonal eosinophilic proliferation subsequent to primary abnormalities in the hematopoietic stem cells. There are three diagnostic criteria for HES. Firstly, persistent eosinophilia of >1.5×10^9^ cells/l. Secondly, the exclusion of secondary causes of eosinophilia, including allergic reactions or parasitic infection, and finally organ damage (5). In addition, according to World Health Organization’s classification of myeloid neoplasm and acute leukemia, the diagnosis of HES requires the exclusion of other acute or chronic myeloid neoplasms (6). The conversion of HES to acute leukemia is infrequent.

Eosinophilic cystitis associated with HES is a rare clinical entity. To the best of our knowledge, only five cases have been reported in the literature (3,7–10). The most common symptoms caused by eosinophilic cystitis include dysuria, gross hematuria and suprapubic pain during micturition (11). However, the mechanisms underlying how and why these overproduced eosinophils infiltrate the bladder and cause the specific symptoms remain unclear. It has been hypothesized that eosinophilic tissue damage is associated with the release of major basic protein, eosinophil peroxidase, eosinophil cationic protein and eosinophil-derived neurotoxin (8). In imaging examinations, the bladder wall exhibits diffuse or localized thickening. The characteristic observation with cystoscopy is erythematous edema, which may be easily mistaken as an invasive tumor or glandular cystitis. Thus, tissue biopsy is essential to establish a definite diagnosis and eliminate possible concomitant bladder malignancies.

Due to the limited number of HES cases, there is no standard guideline therapy for the management of HES and there is no known cure for eosinophilic cystitis. Thus, individualized treatment and close follow-up examinations are essential. The aim of therapy should be to reduce the excessive production of eosinophils in order to prevent organ damage (10). As indicated by the majority of reported cases, prednisone is recommended as the primary drug. Symptomatic patients should be treated with prednisone therapy (1 mg/kg per day) until clinical improvement occurs, followed by a slow tapering regimen of prednisone. The majority of patients experience complete or partial remission following corticosteroid treatment. For symptomatic patients with intracrable disease which is non-responsive to steroids, chemotherapeutic agents, including hydroxyurea, vincristine, 6-mercaptoputine, busulfan and chlorambucil, should be administered. In the present case, partial hematological remission was achieved following prednisone therapy. However, the bladder infiltration was resolved completely. Therefore, we hypothesize that prednisone is an effective agent in reducing organ eosinophilic infiltration and preventing organ damage.

In conclusion, the present study has described a case of HES with eosinophilic cystitis as the initial manifestation. Although this is an uncommon inflammation condition, eosinophilic cystitis should be considered when encountering an HES patient with urinary symptoms.
